# A putative role for amino acid permeases in sink-source communication of barley tissues uncovered by RNA-seq

**DOI:** 10.1186/1471-2229-12-154

**Published:** 2012-08-30

**Authors:** Stefan Kohl, Julien Hollmann, Frank R Blattner, Volodymyr Radchuk, Franka Andersch, Burkhard Steuernagel, Thomas Schmutzer, Uwe Scholz, Karin Krupinska, Hans Weber, Winfriede Weschke

**Affiliations:** 1 Leibniz-Institut für Pflanzengenetik und Kulturpflanzenforschung (IPK), Gatersleben, D-06466, Germany; 2 Christian-Albrechts-Universität (CAU), Kiel, D-24118, Germany

**Keywords:** Barley, Vegetative organs, Developing grains, N remobilisation, N accumulation, RNA-seq, Cysteine peptidases, N transporter genes, Source-sink communication

## Abstract

**Background:**

The majority of nitrogen accumulating in cereal grains originates from proteins remobilised from vegetative organs. However, interactions between grain filling and remobilisation are poorly understood. We used transcriptome large-scale pyrosequencing of flag leaves, glumes and developing grains to identify cysteine peptidase and N transporter genes playing a role in remobilisation and accumulation of nitrogen in barley.

**Results:**

Combination of already known and newly derived sequence information reduced redundancy, increased contig length and identified new members of cysteine peptidase and N transporter gene families. The dataset for N transporter genes was aligned with N transporter amino acid sequences of rice and Arabidopsis derived from Aramemnon database. 57 *AAT*, 45 *NRT1/PTR* and 22 *OPT* unigenes identified by this approach cluster to defined subgroups in the respective phylogenetic trees, among them 25 *AAT*, 8 *NRT1/PTR* and 5 *OPT* full-length sequences. Besides, 59 unigenes encoding cysteine peptidases were identified and subdivided into different families of the papain cysteine peptidase clade. Expression profiling of full-length *AAT* genes highlighted amino acid permeases as the group showing highest transcriptional activity. *HvAAP2* and *HvAAP6* are highly expressed in vegetative organs whereas HvAAP3 is grain-specific. Sequence similarities cluster HvAAP2 and the putative transporter HvAAP6 together with Arabidopsis transporters, which are involved in long-distance transfer of amino acids. HvAAP3 is closely related to AtAAP1 and AtAAP8 playing a role in supplying N to developing seeds. An important role in amino acid re-translocation can be considered for HvLHT1 and HvLHT2 which are specifically expressed in glumes and flag leaves, respectively. PCA and K-means clustering of *AAT* transcript data revealed coordinate developmental stages in flag leaves, glumes and grains. Phloem-specific metabolic compounds are proposed that might signal high grain demands for N to distantly located plant organs.

**Conclusions:**

The approach identified cysteine peptidases and specific N transporters of the AAT family as obviously relevant for grain filling and thus, grain yield and quality in barley. Up to now, information is based only on transcript data. To make it relevant for application, the role of identified candidates in sink-source communication has to be analysed in more detail.

## Background

In crop plants more than 70% of seed nitrogen is remobilised and translocated from vegetative tissues such as stems and senescing leaves [1]. Remobilisation of N follows different time courses, and contributions of various organs and tissues to N economy of developing seeds differ [2]. In cereals, flag leaves and glumes maintain their metabolic activity longer than other vegetative tissues, and their contribution to the final grain yield is high [3].

Up to 75% of reduced nitrogen in photosynthetically active leaf cells is located in the chloroplasts. Ribulose-1,5-bisphosphate carboxylase/oxygenase (Rubisco) represents the major fraction of chloroplast nitrogen [4]. Before nitrogen is exported to the phloem, Rubisco must be degraded to peptides and amino acids [5]. Gene expression analysis in wheat and barley identified several cysteine protease genes with enhanced transcript levels during leaf senescence [6-8]. Certain C1A-type (papain-type) cysteine proteases and possibly also S10-type serine carboxypeptidases are involved in bulk degradation of stromal proteins during leaf senescence [8]. Both types of proteases are potentially synthesised at the endoplasmatic reticulum and channelled by the secretory pathway, which suggests routing to the lytic vacuolar compartment such as small senescence-associated vacuoles [9]. High expression and strong upregulation of genes encoding papain-like cysteine peptidases suggests an important role for especially those family members in naturally senescing barley leaves between 7 and 21 DAF [7].

During senescence, cellular proteins are degraded into peptides and amino acids. Efficient partitioning of amino acids or peptides within the plant requires active transporters to transfer N compounds across cellular membranes [10]. For plants with fully sequenced genomes (e.g. Arabidopsis and rice), the Aramemnon database [11,12] provides annotation and further information for the complete collection of putative N transporter genes, whereas to date only four sequences for putative barley N transporters are listed. Based on sequence similarity, amino acid transporters were grouped into members of the ATF (amino acid transporter family) and the APC (amino acid-polyamine-choline) families. The ATF family can be further divided into AAPs (general amino acid permeases), LHTs (lysine-histidine transporters), proline transporters (ProTs) as well as into transporters with substrates like γ-aminobutyric acid (GATs), aromatic and neutral amino acids (ANTs) and indole-3-acetic acid (AUXs) [10,13,14]. Subdivision of the APC family reveals transporters for cationic (CATs) and L-type amino acids (LATs), as well as the GABA permeases (GAP). Overall 63 (Arabidopsis) and 80 (rice) candidates cluster into these groups.

Peptide transport in plants is accomplished by two gene families, the oligopeptide transporters (OPTs) transporting tetra- and pentapeptides and transporters for di- and tripeptides belonging to the nitrate/peptide transporter family (NRT1/PTR) [14]. In Arabidopsis and rice, 53 and 81 members belong to this group, while 9 and 8 transporters are annotated as OPTs. Whereas a relatively high number of Arabidopsis amino acid and NRT1/PTR transporters are functionally characterized (for reviews see [10,13]) the information for monocots, especially for barley is scarce. The best characterised monocot peptide transporter is HvPTR1 localized in the scutellum of barley grains and responsible for mobilisation of peptides from endosperm into germinating embryos [15,16]. OsPTR6 was shown to transport Gly-His-Gly [17]. From the OPT family, only one monocot sequence (OsGT1) has been functionally characterised so far [18].

Numerous transporters contributing to iron trafficking in plants are described and were functionally characterised for grasses. This is due to the fact that grasses evolved a distinct mechanism to acquire Fe from the soil best described as ‘chelation’ strategy [19]. Strong Fe chelators called phytosiderophores (PS), are synthesised by the plant and secreted into the rhizosphere, where they bind Fe(III). The Fe(III)-PS complex is than taken up by Fe(III)–PS uptake proteins [20,21] called Yellow Stripe-Like (YSL) transporters. Several YSL transporters have been identified and characterised (see for instance [22-25]), among them the barley transporters HvYSL5 [26], HvYSL2 [25] and HvYS1 [27]. The role of YSL transporters in remobilisation and grain filling is unclear yet. YSL transporters are distantly related to the OPT family [28]. In Arabidopsis and rice, 8 and 18 sequences belong to the YSL group.

Although numerous plant amino acid and peptide transporters have been identified and some of them functionally characterised, it is difficult to determine which are the most important for plant N recycling on both the source and the sink side. For barley, this situation is even more complicated as the genomic sequence is only partially assembled [29]. Furthermore, only 0.06% (264 ESTs) from 444,652 barley ESTs in assembly 35 of HarvEST:Barley v1.83 (H35, [30]) represent sequences expressed in glumes and those 33,376 ESTs (7.47%) derived from leaf cDNA libraries are not representative for remobilising flag leaves. Sequence information from EST collections might also be reduced for membrane-associated compounds because of high instability of respective *E. coli* clones.

Next generation sequencing (NGS) technologies offer new opportunities to analyse plants without fully sequenced genomes. Transcriptome large-scale parallel pyrosequencing was addressed to flag leaves, glumes and developing grains in order to analyse remobilisation and import of N compounds immediately before and after seed set. Data evaluation was focussed on two specific groups of genes responsible for remobilisation and accumulation of nitrogen, cysteine peptidases and N transporters. Combination of publicly available and RNA-seq data reduced redundancy, increased length of gene-specific contigs and identified new members within the respective gene families. Members of the *AAT* gene family were over-represented in the set of RNA-seq N transporter sequences. Sequence alignment allowed to reconstruct 25 full-length *AAT* genes. Based on temporal expression profiling of these genes we hypothesise that establishment of high N-sink strength in developing grains is perceived in flag leaf and glumes, the tissues in close proximity to developing seeds. We postulate that metabolites communicate the increasing sink strength to the remobilising tissues by modulating transcript amounts as shown here for amino acid permeases. Thus, *AAT* gene activities might be involved in source/sink communication in barley. In addition, fluctuating transcript abundances of *AAT* genes especially in flag leaves might reflect tissue-specific regulation of sink/source transition.

## Results

### RNA-seq and sequence assembly

mRNA was prepared from barley flag leaves, glumes and caryopses collected at different stages of grain development. Equal amounts of RNA were combined from each stage at 2 day intervals, from 4 days before anthesis up to 24 days after flowering (DAF) for flag leaves and glumes, between anthesis and 24 DAF for caryopses.

After quantification and quality control of the samples, reverse transcription and normalisation of the three libraries as well as transcriptome sequencing was performed by GATC Biotech (Konstanz, Germany). One half Roche/454 GS-FLX run was performed for each library. From a total of 1,806,025 reads 701,026 distribute to flag leaves (FL), 557,505 to glumes (GL) and 547,494 to grains (G). Adaptor and quality trimming reduced read-yield to 1,585,811 (615,568 + 485,800 + 484,443, see Table 1). Average read length was 397 bp (FL), 400 bp (GL) and 392 bp (G). For each organ, reads were clustered and assembled into contigs and singletons (Table 1). Flag leaves showed highest contig number (43,467) but lowest contig length (688 bp). Glumes revealed lowest contig number (31,022) and highest contig length (835 bp). Grains showed moderate levels (37,790 and 791, Table 1). On average, each contig was covered by 11 to 15 reads. Whereas contig numbers were comparable between the three organs, numbers of singletons vary from high values in FL and G (97,348 and 82,446) to low values in GL (29,388). This suggests high numbers of lowly expressed genes in flag leaves and grains and low numbers of highly expressed genes in glumes, indicating lower complexity of the glumes transcriptome.

**Table 1:** Output of large scale RNA-seq and sequence assembly

	**Flag leaf**	**Glumes**	**Grains**
**Total reads**	615,568	485,800	484,443
**Ø Read length**	397	400	392
**Total contigs**	43,467	31,022	37,790
**Ø Reads/contig**	12	15	11
**Ø Contig size**	688	835	791
**Total singletons**	97,348	29,388	82,446

### Annotation

Contigs and singletons obtained after sequence assembly were characterised by a multilevel process of filtering from barley-specific to more general data sets as well as from high to low stringency using the different databases in the following order: (1) H35 [30], (2) UniProtKB/Swiss-Prot [31], (3) UniProtKB/TrEMBL [31] and (4) non-redundant dataset from NCBI [32] with E-value cut-offs of <1E^-20^, <1E^-20^, <1E^-10^ and <1E^-5^, respectively. Sequences without a match in one database were compared to the next one. The output of annotation is given in Table 2.

**Table 2:** Annotation of RNA-seq contigs and singletons

**BLASTn vs**	**Flag leaf**	**Glumes**	**Grains**
	**No. of contigs**		
HarvEST35 (e^-20^)	33,743	24,416	30,077
UniProtKB/Swiss-Prot (e^-20^)	1,196	542	635
UniProtKB/TrEMBL (e^-10^)	3,573	2,183	2,302
non-redundant (e^-5^)	754	386	453
Total hits	39,266	27,527	33,467
Total no hits	4,201	3,495	4,323
	**No. of singletons**		
HarvEST35 (e^-20^)	38,119	16,200	32,390
UniProtKB/Swiss-Prot (e^-20^)	843	509	611
UniProtKB/TrEMBL (e^-10^)	5,809	2,840	4,892
non-redundant (e^-5^)	4,986	887	3,992
Total hits	49,757	20,436	41,885
Total no hits	47,591	8,952	40,561

Percentages of total contig hits are comparable between glumes and grains (88.73% and 88.56%) but are higher for flag leaves (90.34%). BLAST searches against the different databases revealed 14.1% (FL), 11.3% (GL) and 10.1% (G) of new contig sequences as not functionally described in H35. For the total no hit category, results for flag leaves are different from those of glumes and grains (9.7%, 11.3% and 11.4%, respectively). Percentages of total-hit singletons are comparable for flag leaves and grains (51.1% and 50.8%) but higher for glumes (69.5%). This result coincides with the observed low number of glumes singletons (Table 1) and indicates lower complexity of the glumes transcriptome compared to flag leaves and grains.

The sum of contigs and singletons annotated from flag leaves (33,743 + 38,119) yields 71,862 expressed genes, which is clearly higher than the number of unigenes from H35 (50,938). This may reflect high redundancy of the flag leaf dataset, which is also obvious for grains (62,467 annotated sequences) but not for glumes (40,616 annotated sequences). This can be explained by the fact that only RNA-seq reads were used for the assembly process. Obviously, some of the RNA-seq singletons and/or contigs represent the same gene, but do not overlap and thus increase the numbers within the total hit category. The contribution of contigs should be lower than that of singletons.

### Tissue-specificity of contigs

**Figure 1:**
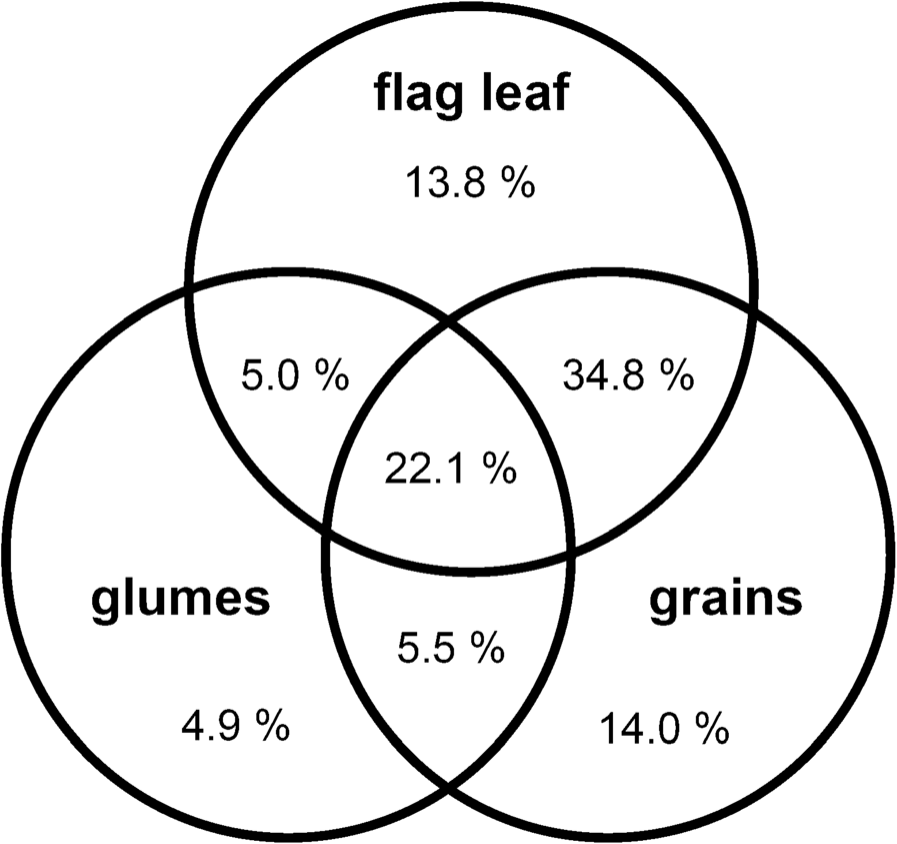
Venn diagram showing tissue specificity of the CAP3-contigs.

Figure 1 shows tissue-specificity of CAP3 contigs. The percentage of contigs expressed exclusively in glumes (4.9%) is remarkably lower than that expressed specifically in flag leaves (13.8%) or grains (14.0%). The transcriptome of the glumes contains a low number of sequences identical with those expressed in flag leaves (5.0%) and grains (5.5%). In contrast, a high percentage of sequences (34.8%) is shared between flag leaves and grains.

**Figure 2:**
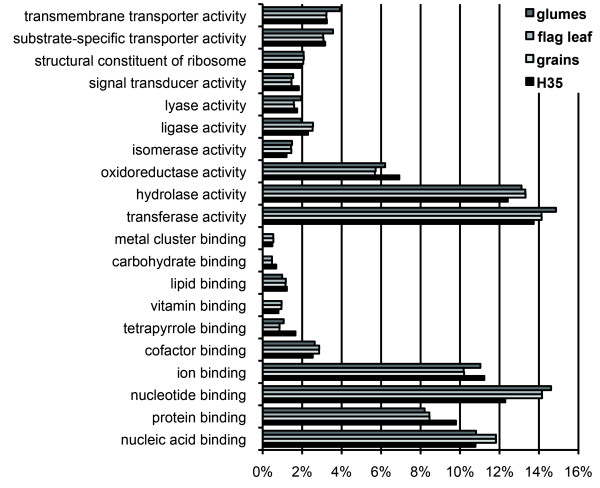
**Blast2GO annotation of RNA-seq contigs and H35 unigenes.** The result is based on gene ontology terms level 3 of the category Molecular Function. 21,525 sequences from flag leaves, 10,361 sequences from glumes, 21,199 sequences from grains and 22,043 sequences from the H35 database were annotated.

For an overview on the tissue specificity of molecular functions, pyrosequence contigs and H35 unigenes were annotated based on gene ontology terms [33] and analysed by Blast2GO software [34,35]. The results from level 3 of the category Molecular Function are depicted in Figure 2.

There are two main findings: (i) flag leaf and grain transcriptomes are highly similar in all depicted categories and (ii) the glumes transcriptome differs from flag leaves and grains, but is comparable to H35. Categories “trans-membrane transporter activity”, “substrate-specific transport activity” and to lesser extent “transferase-activity” and “nucleotide binding” are enriched in glumes. Especially for “ion binding”, “nucleic acid binding”, “lyase and ligase activities”, “oxidoreductase activity” and “cofactor binding”, the glumes transcriptome is more similar to that of H35 than to those of flag leaves and grains.

### RNA-seq gained new sequence information for N transporters and cysteine peptidases

**Figure 3:**
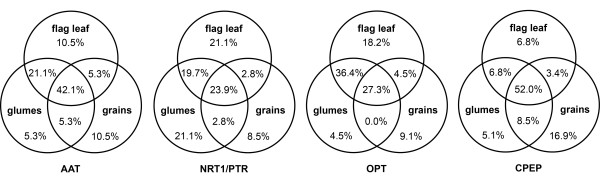
Venn diagrams showing organ-specific expression of N transporters and cysteine peptidases.

Based on annotation, sequences encoding putative N transporters and cysteine peptidases were selected from H35 unigenes and independent BLAST searches were performed within RNA-seq contigs. Combining H35 and RNA-seq information, new sets of N transporter and cysteine peptidase unigenes were created and manually revised for each tissue (tissue-specificity is summarised in Figure 3).

**Figure 4:**
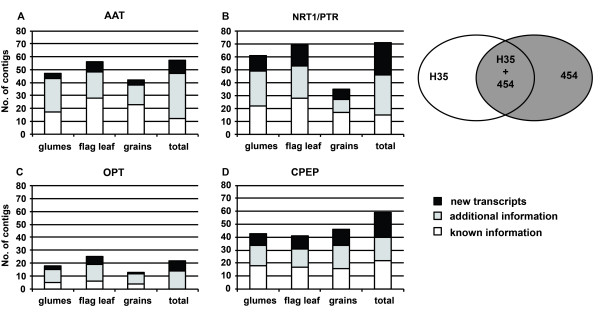
**New sequence information for N transporter and cysteine peptidase genes.** Comparison of RNA-seq and H35 unigenes for (**A**) *AAT*, (**B**) *NRT1/PTR* and (**C**) *OPT* transporter sequences and for (**D**) cysteine peptidase unigenes. New information (black areas) from RNA-seq shows less than 98% identity to H35 unigenes at amino acid level. Additional information (gray-shaded) matches H35 unigenes, but extends information by more than 50 bp or closes gaps between unigenes. Known information (white areas) did not add new knowledge. Origin of sequences is represented by gray-shaded areas of the overlapping ellipses at the right side.

To evaluate the power of the RNA-seq approach for N transporter and cysteine peptidase gene families, the contigs were compared with H35 (Figure 4). After combining information from all three organs, between 67% (CPEP) to 100% (OPT) of pyrosequencing contigs extend known or add new sequence information in comparison to H35.

**Figure 5:**
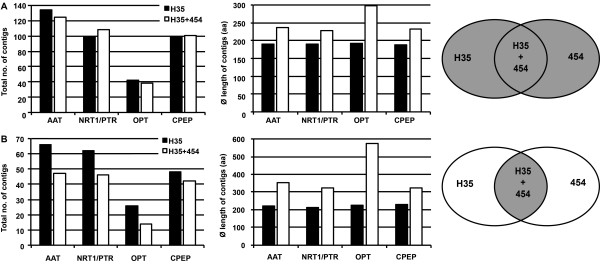
**Number and average length of unigenes encoding putative N transporters and cysteine peptidases.** (**A**) Total number and average length of contigs after assembly of all N transporter and cysteine peptidase unigenes available from RNA-seq and H35. (**B**) Number and length of N transporter and cysteine peptidase contigs containing sequence information overlapping between the two sources. Black bars represent H35 information, white bars show results after combining H35 and RNA-seq unigenes. Gray-shaded areas in the ellipses at the right-hand side represent the origin of the sequences.

Results from assembling all candidate sequences from the three libraries and H35 are summarised in Figure 5A and show a clearly increased average contig length (right panel), whereas for total contig number no general tendency was found (left panel). In the subset of contigs containing information from both sources (Figure 5B) the contig number is reduced for all gene groups and the increase in average contig length is more pronounced than for the overall dataset.

### RNA-seq contigs of N transporters and cysteine peptidases cluster with annotated homologs from Arabidopsis and rice and enlarge available full-length sequences

#### Papain-like cysteine peptidases

For all further analyses, sequences derived from the combination of pyrosequencing and H35 as well as unique pyrosequences were considered.

59 genes encoding putative papain-like cysteine peptidases of the CA clan were assembled (Table 3). Based on sequence similarities, the resulting contigs were subdivided into different families of the CA clan (Merops database, [36,37]). In general, numbers of barley unigenes defined after sequence assembly were lower than numbers of full-length genes in Arabidopsis and rice except for the families C02, C85 and C88. The C02 cysteine peptidase of Arabidopsis and rice is encoded by a single copy gene. In contrast, two full-length genes and one additional contig were identified for barley, reflecting at least three gene family members. Despite the unclear situation for C85, this subfamily might harbour promising candidates for further investigation, as the high contig number suggests implication for this type of cysteine peptidases in remobilisation processes taking place in at least one analysed tissue.

**Table 3:** Putative papain type cysteine peptidases in different plant species

**Clan**	**Family**	H.v.*	***A.t.***	***O.s.***
	C01	26 (15/4)	40	51
	C02	3 (2/1)	1	1
	C19	7 (1/1)	37	22
CA	C54	1 (0/0)	2	2
	C65	1 (0/0)	1	3
	C85	17 (2/2)	6	7
	C88	4 (1/0)	1	2
	total	59	88	88

* full-length barley sequences are given in brackets (total number/new from RNA-seq).

#### N transporters

The new dataset was aligned with all N transporter amino acid sequences of rice and Arabidopsis derived from Aramemnon [12] and phylogenetic trees were constructed.

**Figure 6:**
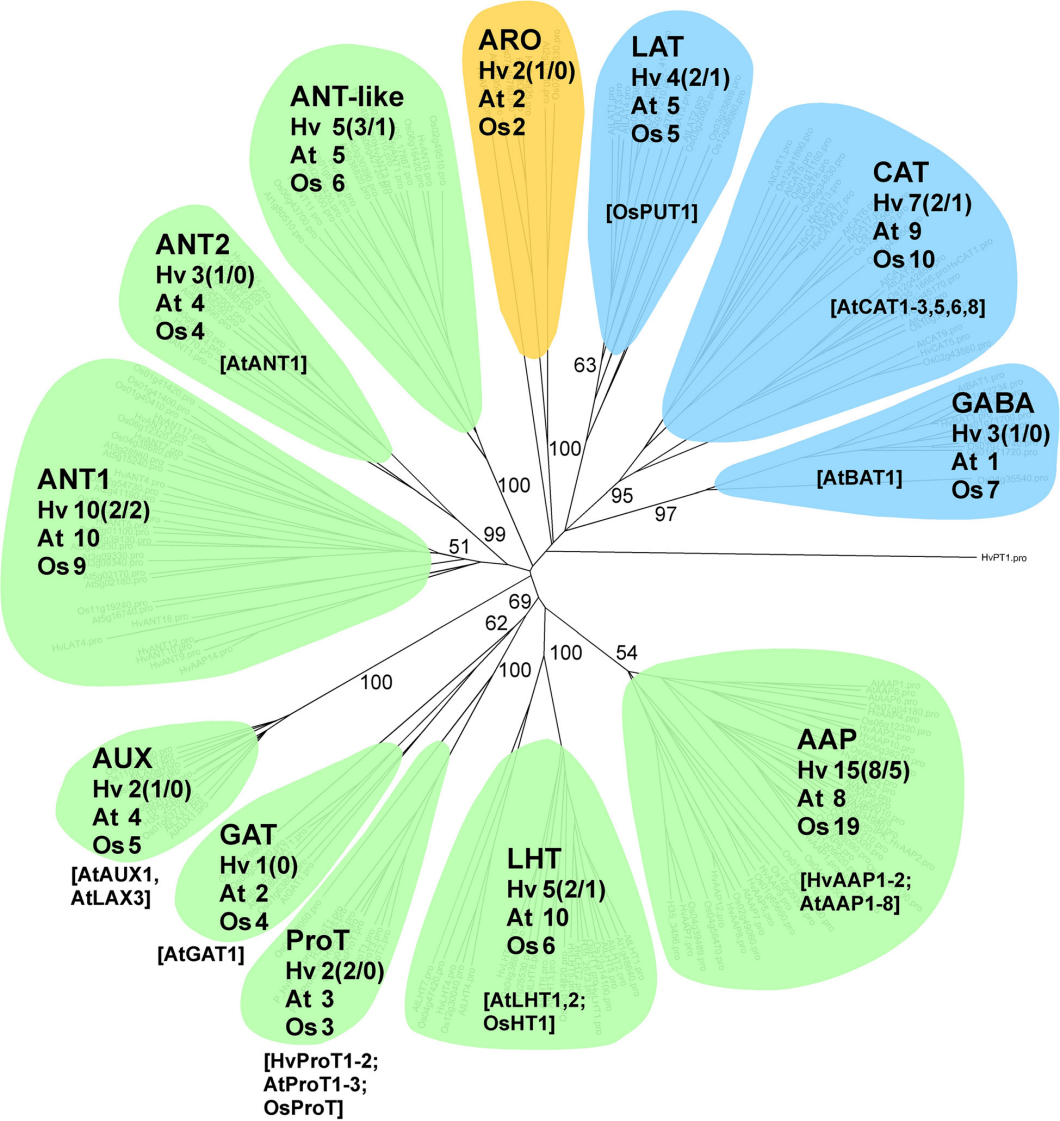
**Phylogenetic tree of plant AATs.** Clustering of 63 Arabidopsis, 80 rice and 59 unique barley sequences with *H. vulgare* phosphate transporter 1 (HvPT1) as outgroup. Colours indicate membership to different subgroups of ATF (green) and APC (blue) families, members of the aromatic amino acid transporters are shown in orange. Full-length barley sequences are given in brackets (total number/new from RNA-seq), functionally characterized transporters are given in square brackets and mentioned in Additional file 2: Table S2. Sequences from Arabidopsis and rice, including their respective nomenclature, were extracted from Aramemnon, barley sequences derived from RNA-seq, H35 (only full-length sequences), publications (HvProT, HvProT2) and previous unpublished work (HvAAP1 + 2) - see also Additional file 2: Table S2. The phylogenetic tree was constructed using the neighbor-joining algorithm in the program PAUP* [78]. The tree was displayed and manipulated using FigTree [79]. Clustering of AAT sequences into different subgroups is supported by the sequence distance matrices (Additional file 3: Figure S2). Detailed version of the phylogenetic tree including ID-numbers of all sequences is given in Additional file 1: Figure S1.

All putative members of the AAT family from barley cluster into the subgroups described for *A. thaliana* by Rentsch et al. [13]. We furthermore included the group of transporters related to EcTyrP, a tyrosine-specific permease of *E. col*. [38]. The phylogenetic tree of AAT sequences (Figure 6, Additional file 1: Figure S1) contains 33 functionally characterised transporters (Additional file 2: Table S2). 26 of them reside in the ATF clade and seven are APC transporters. Most genes with proven functionality (27) are from Arabidopsis, only three from rice, and four from barley. These transporters are listed in Additional file 2: Table S2 and include two barley transporters (HvAAP1 and 2) that were characterised in the author’s lab.

From 71 RNA-seq contigs of the barley NRT1/PTR family only 45 cluster into the four subfamilies defined by Tsay et al. [14], while 26 sequences form a separate branch (data not shown). As BLAST analysis showed no obvious differences between these 26 and the other 45 candidates in sequence similarity to the clustering rice sequences (data not shown), a unique group in barley seems unlikely and these sequences were omitted from the tree. This deviant behaviour might be explained by the overall heterogeneity within this group or the limited sequence information of these outliers (average length of 184 aa compared to 305 aa for sequences that clustered).

**Figure 7:**
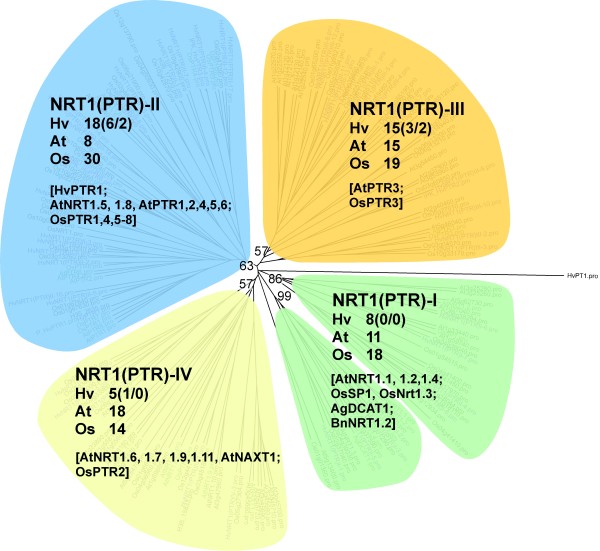
**Phylogenetic tree of plant NRT1/PTRs.** Clustering of 52 Arabidopsis, 81 rice and 46 unique barley sequences; for consolidation of the tree, sequences from *Alnus glutinosa* (AgDCAT1) and *Brassica napus* (BnNRT1) were included (according to Tsay et al. [14]). Colours indicate membership to subgroups I (green), II (blue), III (orange) and IV (yellow) as defined by Tsay et al. [14]. Barley sequences were derived from RNA-seq, H35 (only full-length sequences), publications (HvPTR1) and previous unpublished work (IPK_HvPTR2, 3, 6). Clustering of NRT1/PTR sequences into different subgroups is supported by the sequence distance matrices (Additional file 5: Figure S4). Detailed version of the phylogenetic tree including ID-numbers of all sequences is given in Additional file 4: Figure S3. For further explanations see legend of Figure 6.

According to Tsay et al. [14] the subgroups are named NRT1/PTR-I, II, III and IV (Figure 7, Additional file 4: Figure S3) although our data would suggest an adjustment of this classification, as the members of the NRT1/PTR-I group are not monophyletic. The high heterogeneity within subgroup I is also reflected by the sequence distance matrix data (Additional file 5: Figure S4) showing similarities of 39.1% for subgroup I in comparison to 44.6% and 42.3% for subgroup II and III, respectively. Although percentages of similarity are even lower for subgroup IV (37.9%), this branch is monophyletic confirming the basic structure presented by Tsay et al. [14]. 30 of the presented NRT1/PTR sequences are functionally characterised (Additional file 2: Table S2), 18 of them are coming from Arabidopsis, eleven from rice. From barley, only HvPTR1 belonging to subgroup II is functionally characterised so far [15].

**Figure 8:**
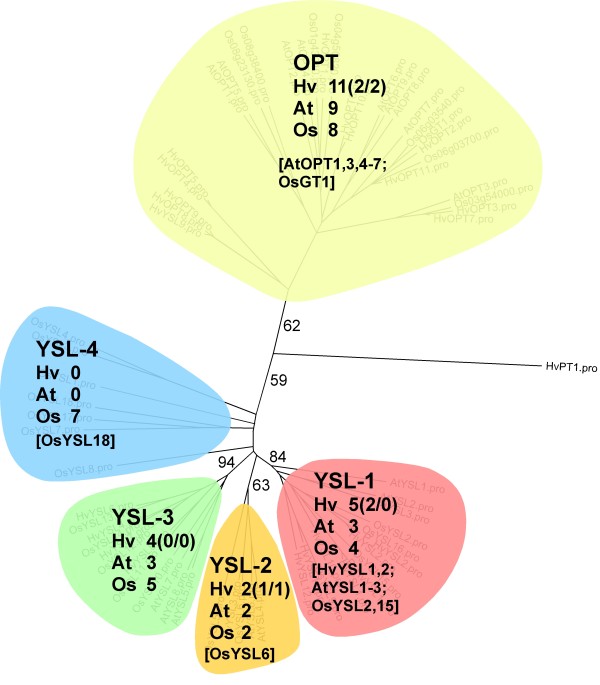
**Phylogenetic tree of plant OPTs.** Clustering of 17 Arabidopsis, 26 rice and 22 unique barley sequences. Colours indicate membership to the OPT (yellow) and the yellow stripe-like (YSL) family. According to Zheng et al. [26] YSL transporter sequences are subdivided into the subgroups YSL-1 (red), YSL-2 (orange), YSL-3 (green) and YSL-4 (blue). Barley sequences were derived from RNA-seq and publications (HvYSL1 + 2). Clustering of OPT sequences into different subgroups is supported by the sequence distance matrices (Additional file 6: Figure S6). Detailed version of the phylogenetic tree including ID-numbers of all sequences is given in Additional file 7: Figure S5. For further explanations see legend of Figure 6.

All 22 barley sequences functionally annotated as putative OPT transporters cluster into the phylogenetic tree presented in Figure 8. Phylogenetic analysis clearly separates sequences of the OPT type from yellow stripe-like (YSL) transporters. Also the classification within the YSLs according to Zheng et al. [26] was reproduced. Transporters of the YSL groups 1, 2 and 3 show a high degree of sequence similarity whereas the YSL-4 group is more diverse (Additional file 6: Figure S6) and not monophyletic (Figure 8, Additional file 7: Figure S5). Furthermore, this group is rice specific with only OsYSL18 functionally characterised [24]. NGS transcriptome data from the rice genome annotation project [39] point to pollen-specificity of this YSL-transporter group. Sequences of putative HvYSL-transporters belonging to subgroups 1, 2 and 3 might be starting points to analyse micronutrient transport into developing barley grains. The highest number of functionally characterised OPTs resides within the OPT and the YSL-1 groups (10 and 8 sequences) while no YSL-3 transporter is functionally characterised so far.

### Additional contribution from sequence analysis of barley full-length cDNAs

The H35 and pyrosequencing output was compared with 24,783 barley full-length cDNAs [40]. Combining information from these datasets reduced the number of RNA-seq unigenes for all three N transporter classes and *CPEP* genes (see Table 4), but also revealed redundancy within the full-length cDNA approach. Thus, a fraction of these sequences are redundant, at least with regard to N transporters and *CPEP genes*.

**Table 4:** Comparison of N transporter and cysteine peptidases (CPEP) sequence information from full-length cDNA* and RNA-seq data

**Gene family**	**Raw data**		**After assembly**		**Found in H35**		**New**				**Overall**
	**cDNA**	**RNA- seq**	**cDNA**	**RNA- seq**	**cDNA**	**RNA- seq**	**cDNA**	**Excl.**	**RNA- seq**	**Excl.**	
**AAT**	99	57	69	55	54	46	15	9	9	3	78
**NRT1/PTR**	98	71	55	56	42	42	13	8	14	9	71
**OPT**	27	22	19	20	14	14	9	8	6	5	29
**CPEP**	120	59	89	59	78	40	11	11	19	19	120

*Matsumoto et al. [40]; numbers of unigenes shown.

Both approaches identified additional and so far unknown sequences. Matsumoto et al. [40] identified 36 novel putative N transporter and *CPEP* genes, 36 novel genes were detected by pyrosequencing. In summary, sequence information of expressed barley N transporters and cysteine peptidases comprises 78 *AAT*, 71 *NRT1/PTR*, 29 *OPT* and 120 *CPEP* unigenes (Table 4). All RNA-seq unigenes can be considered as expressed in flag leaves, glumes or grains and are barley-specific as checked against the barley genome sequence [41].

### Expression profiling of AAT genes revealed coordinate distinct developmental stages in flag leaves, glumes and grains

RNA-seq and H35 sequences were assembled into 57 *AAT*, 71 *NRT1/PTR* and 22 *OPT* unigenes (Table 5) and, except 26 NRT1/PTR sequences, they cluster into defined subgroups in the respective phylogenetic trees (Figures 6, 7, 8, Additional file 1: Figure S1, Additional file 4: Figure S3, Additional file 7: Figure S5). This unigene set represents 25 *AAT*, 8 *NRT1/PTR* and 5 *OPT* full-length sequences and shows a considerably higher percentage of full-length *AAT* genes compared to putative *NRT1/PTR* and *OPT* genes (42.3% versus 15.7% and 22.7%, respectively). To exclude a predominant bias of H35 leading to these numbers, we compared RNA-seq read numbers contributing to *AAT*, *NRT1/PTR* and *OPT* contigs. Table 5 shows the number of *AAT*, *NRT1/PTR* and *OPT* reads representing a ratio of 3:2:1. After normalising read numbers against the average length of annotated rice N transporters a ratio of 4.0:2.3:1.0 is obtained (data not shown). So both, the raw and normalised read numbers point to higher transcriptional activity of *AAT* genes in comparison to that of the *NRT1/PTR* and *OPT* group. Because of their over-representation in the set of RNA-seq N transporter sequences we suggested that this group of N transporters might play an important role in N retranslocation and grain filling. Because remobilisation related to increasing grain sink strength might be reflected by changing transcript levels of associated N transporters we decided to compare expression of the full-length *AAT* genes in the three organs.

**Table 5:** Overview of sequence information on N transporter genes

**N transporter group**	**Reads**	**Unigenes**	**Full-length**	**% full-length**
**AAT**	2,973	57	25	42.3
**NRT1/PTR**	2,040	71	8	15.7
**OPT**	1,016	22	5	22.7

**Figure 9:**
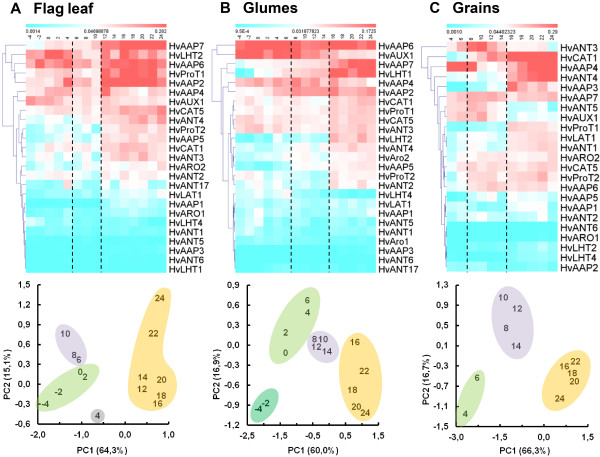
**Transcript profiling, principle component analysis (PCA) and K-means clustering of 25 AAT genes.** Distinct developmental phases were identified in flag leaves (**A**), glumes (**B**) and developing grains (**C**). Tissues were analysed in two-day steps starting 4 days before anthesis (-4) in flag leaves and glumes and at 4 DAF in developing grains until 24 DAF. The heat maps (upper panels) reflect relative transcript abundances after normalisation against *actin* expression (blue = low expression; red = high expression). Developmental phases as identified by PCA are given in the lower panels, numbers represent DAF. Results of K-means clustering are visualized by encircling of respective stages. Light violet areas represent the transition phase.

qRT-PCR analysis was used to estimate transcript amounts in two-day intervals starting 4 days before anthesis in vegetative tissues and at anthesis in grains until 24 DAF, when grain desiccation starts (Figure 9, upper panel). Among the group of 6 *AAT* genes showing highest expression in each of the three organs, *AAPs* are clearly overrepresented (Table 6). Besides, LHT transporters seem to be important for remobilisation. *HvLHT2* and *HvLHT1* are highly expressed in flag leaves and glumes, respectively. In developing grains their expression is either very low or not detectable. There, specific members of the ANT group are highly expressed, *HvANT3* between 6 and 8 DAF and *HvANT4* during early as well as late grain development (Figure 9, upper panel).

**Table 6:** *AAT *genes with high expression in flag leaves, glumes and developing grains^*^

	**Flag leaf**	**Glumes**	**Grains**
**AAP**	*HvAAP2*	*HvAAP2*	*-*
	*-*	*-*	*HvAAP3*
	*HvAAP4*	*HvAAP4*	*HvAAP4*
	*HvAAP6*	*HvAAP6*	*-*
	*HvAAP7*	*HvAAP7*	*HvAAP7*
**others**	*-*	*HvLHT1*	*-*
	*HvLHT2*	*-*	*-*
	*HvProT1*	*-*	*-*
	*-*	*HvAUX1*	*-*
	*-*	*-*	*HvCAT1*
	*-*	*-*	*HvANT3*
	*-*	*-*	*HvANT4*

^*^For each tissue the six AAT genes showing highest expression levels are presented.

To visualise relationships between the three tissues, principle component analysis (PCA) was applied to the tissue-specific qRT-PCR results. Then, K-means clustering was used to identify developmental stages that might be related to each other. The results of the two-step procedure are depicted in the lower panels of Figure 9. In each panel, coloured areas represent related stages. For all three organs, a group including stages 8 and 10 DAF was identified (violet areas). Besides, developmental stages representing the late phase of grain development form separate groups (areas coloured in yellow). K-means clusters coloured in green represent stages of early development. They are highly dispersed, especially between flag leaves and glumes (lower panels of Figure 9A, B).

## Discussion

The majority of N accumulating in cereal grains originates from proteins remobilised from vegetative organs, but interactions of grain filling and remobilisation are only poorly understood. Here we used large-scale transcriptome pyrosequencing of flag leaves, glumes and developing grains to identify putative cysteine peptidases and transporters of amino acids, peptides and oligopeptides involved in N remobilisation and retranslocation into developing grains. This approach suggests that distinct amino acid transporters might be important in sink-source communication between remobilising organs and accumulating grains.

### RNA-seq revealed the specific character of the glumes transcriptome

The read numbers gained by transcriptome sequencing are similar with about 0.5 million for each organ with higher values for flag leaves (Table 1). Also average length and number of reads per contig are comparable between the three organs. In glumes lower contig numbers but higher read numbers per contig were found and furthermore the number of singletons in the glume transcriptome is only one third compared to flag leaves and grains. This suggests either higher specificity or lower complexity of the glumes transcriptome.

Comparison of the three transcript sets as visualised in Figure 1 revealed high similarity between flag leaf and grain contigs (34.8% of identical sequences). On the other hand, only about 5% of glumes sequences are identical with those in flag leaves or grains, pointing again to either higher specificity or lower complexity of the glumes transcriptome.

Another argument underlining the specific character of the glumes transcriptome comes from annotation of organ-specific RNA-seq contigs and its comparison to H35 based on gene ontology terms (Figure 2). These results suggest a different function of the glumes transcriptome compared to the two other tissues especially regarding transport activity. Potential functions of the glumes transcriptome are more similar to these of H35 than to those of flag leaves and grains. H35 contigs consist of ESTs derived from several different tissues. Thus, functional annotation of the glumes transcriptome points to expression patterns representing an average of many tissues. This indicates that annotated functions in glumes are less tissue-specific, whereas transcriptomes of flag leaves and grains seem to be tissue-specific and functional annotation indicates similarity between the two organs.

In summary, comparisons between the transcriptomes of flag leaves, glumes and grains indicates that gene expression in glumes is less tissue-specific and might be characterised by higher activity of a lower number of genes. The glumes transcriptome seems to be different from those of flag leaves and grains whereas the latter two organs reveal functional similarity to each other.

### Glumes might function as mediator between remobilising vegetative tissues and accumulating grains

Relative to grains, both flag leaves and glumes are source organs. Nitrogen mobilisation during grain filling and the role of flag leaves and glumes have been studied predominantly in wheat [3,42,43]. These studies revealed different cellular organisation and distribution of glumes compared to leaves of the same developmental stage [43]. Glumes have more sclerenchyma cells, which serve as a supporting structure for the grain. Compared to flag leaves, glumes contain less green tissue and, consequently, fewer chloroplasts and less Rubisco [44,45].

During grain development, a decline in the content of soluble proteins is detected in both flag leaves and glumes but patterns of remobilisation differ. Protein content in flag leaves remains constant up to anthesis and declines when grains develop. Glumes continue to accumulate protein until 5 DAF before remobilisation starts. The different initiation time of remobilisation suggests that glumes act as a transient sink for N derived from flag leaves and senescing vegetative organs. These studies indicate that glumes are supplying nitrogen to the grains during later developmental stages [43].

Glumes contain high percentages of Gln, Pro, Lys, Arg and His [43]. Considerable high contents of Gln, Lys, Arg and His also occur in the nucellar projection (NP) compared to endosperm transfer cells (ETC) at the beginning of grain filling [46]. The NP/ETC complex represents the transfer path between maternal and filial grain tissues and also functions as a metabolic interface to precondition amino acid supply to the developing endosperm. In NP cells, gene expression of different cytosolic isoforms of Gln synthetase (GS) could be involved in re-assimilation of ammonia from protein breakdown and production of N transport compounds [46]. Such a function has been suggested also for GS present in glumes [43].

In summary, flag leaves and glumes obviously function differently, at least during early grain development when sink strength of the endosperm is still low. Analogies can be observed between glumes and supplying maternal grain tissues. This suggests that the glumes metabolism is adjusted to the changing demands of developing grains and points to a putative function of the glumes as mediator between (remobilising) vegetative tissues and (accumulating) grains.

### RNA-seq identified a set of putative cysteine peptidase and N transporter genes possibly involved in remobilising and accumulating of nitrogen

RNA-seq provided new sequence information for cysteine peptidases and N transporter genes compared to H35 (Figure 4), reduced redundancy and increased unigene length within H35 data (Figure 5B). With respect to full-length cDNAs published by Matsumoto et al. [40], 36 contigs were identified as unique in the collection of pyrosequences (Table 4). While these new sequences might be involved in degradation and retranslocation of N compounds during grain development those cDNAs present only in the H35 or Matsumoto collections should be less relevant for such functions.

#### Cysteine peptidases

Papain-like cysteine peptidases play an important role in naturally senescing barley tissues [8,9], especially between 7 and 21 days post anthesis [47]. Although several genes encoding cysteine proteases are upregulated during senescence [6,8,48-50], direct evidence for the implication of specific members from this class of proteases in protein degradation is lacking.

Combination of H35 and RNA-seq sequence information identified a set of 59 unigenes that encode Papain-like cysteine peptidases (Table 4). This set represents 21 full-length sequences and 38 unigenes that belong to an unknown number of genes. Some of these sequences might belong to the same gene but cannot be aligned (redundancy problem). These candidates can be considered as active between anthesis and DAF 24 in at least one of the three tissues. In comparison, the total number of cysteine peptidase genes from the same peptidase family of rice and Arabidopsis is high (88 candidate genes in both species) pointing to a certain degree of specificity of the newly assembled contigs for remobilisation. In the C01 and C85 families most unigenes (26 and 17), as well as most new full-length sequences (4 and 2) were found (Table 4), predestining their members as promising candidates. Analysis of tissue-specificity and localisation of the respective gene products to defined cellular compartments remain to be done.

#### Amino acid permeases seem to be predominant in N retranslocation and grain filling

AAPs seem to be predominant in N retranslocation and grain filling. This conclusion was derived from over-representation of this gene family in the set of RNA-seq derived N transporter sequences (Table 5) and from its very strong expression in both, source and sink tissues (upper panel of Figure 9, Table 6). Two of the highly expressed putative *AAP* genes (*HvAAP2, HvAAP6*) are active only in the source tissues flag leaves and glumes, two others (*HvAAP4* and *HvAAP7*) are expressed in source as well as sink tissues. Among the putative transporter genes listed in Table 6, *HvAAP3* is specific for grains. HvAAP3 but also HvAAP4 show high sequence similarity to AtAAP1 and AtAAP8 (Additional file 1: Figure S1). The two Arabidopsis transporters play a role in supplying developing seeds with nitrogen [51,52]. HvAAP2 and HvAAP6 are closely related to AtAAP2 and AtAAP5 (Additional file 1: Figure S1). AtAAP5 is expressed in mature leaves, stems and flowers and involved in long-distance transfer of amino acids, especially glutamine, the predominant amino acid found in the phloem [53]. Promoter-reporter gene fusions showed that *AtAAP2* is expressed in vascular tissues of stems and siliques. Furthermore, *AtAAP*2 expression is tightly associated with phloem strands that connect to fruits. Thus, AtAAP2 seems to be an excellent candidate for xylem-phloem transfer along this path [54,55], a role that might also be assumed for HvAAP2 and HvAAP6. HvAAP7 is a member of a separated branch of the AAT tree harbouring only uncharacterised rice and barley sequences (Additional file 1: Figure S1). Because of its high expression in flag leaves and glumes during grain filling, *HvAAP7* can be considered as being an interesting candidate for functional studies in barley.

Besides members of the AAP family, the two putative transporters HvLHT1 and HvLHT2 seem to be specifically important for N retranslocation in flag leaves (HvLHT2) and glumes (HvLHT1). At the sequence level, the two proteins are closely related to each other and to the functionally characterised OsHT1 [56]. Because of its high expression and strong tissues-specificity (see upper panels of Figure 9), *HvLHT1* might be an excellent candidate to elucidate the specific role of glumes for N supply to the developing grains. Two members of the ANT gene family (*HvANT3*, *HvANT4,* Table 6) are highly expressed in developing grains (Figure 9, upper right panel). Because only one member of the large ANT family is functionally characterised so far (AtANT1, [57]), any hint to possible functions of HvANT3 and HvANT4 in grain filling is missing.

### A putative role for amino acid transporters in sink-source communication

Seed sink strength for N, which means the ability of the grain to attract and import N compounds, is due to high storage protein synthesis and high demand and/or intensity of active uptake via membrane-localised transporters [58]. Recent work in our lab demonstrated that increasing sink strength due to overexpression of an amino acid transporter in legume seeds increases amino acid supply, total seed N and protein content [59,60].

In barley grains, highest expression of storage protein genes occurs between 10 and 12 DAF [61]. Storage protein accumulation starts two days later in aleurone and starchy endosperm cells [62]. Simultaneously, when high N sink strength is initiated a set of AAT genes is transcriptionally activated in grains (Figure 9C, upper panel). Remarkably, expression of these genes is low between 8 and 14 DAF, but higher during early development. PCA and K-means clustering of AAT gene expression data, clearly separate three groups of data points belonging to stages 4 and 6 DAF, 8 to 14 DAF and 16 to 24 DAF (lower panel of Figure 9C). These groups have been assigned to pre-storage, intermediate (transition) and storage phases of barley grain development, respectively. This staging of grain development has been deduced from transcript profiling of 12,000 grain-expressed unigenes. Data evaluation justified the intermediate phase between 6 and 10 DAF [61]. Considering expression profiles of AAT genes alone, the intermediate phase would start two days later and would be prolonged to 14 DAF (lower panel of Figure 9C). This reflects the interval between beginning starch accumulation in the differentiated caryopsis centre (6 DAF, [63]) and high storage protein synthesis in the peripheral parts of the grain. Such difference in the beginning of the transition phase reflects delayed beginning of protein accumulation compared to starch biosynthesis, and also reveals the internal gradient of caryopsis differentiation.

The highly expressed members of the AAT gene family in flag leaves and glumes differ from those in filling grains (upper panels of Figure 9), but phases of grain development are also reflected in the supplying organs flag leaves and glumes (lower panels of Figure 9). In grains and glumes, transition phase and grain filling include the same stages (8 to 14 DAF and 16 to 24 DAF, respectively). This supports the hypothesis that glumes adjust metabolism according to the specific demands of the grains. Remarkably, expression of *AAT* genes in flag leaves is elevated four days earlier than in glumes and grains (upper panels of Figure 9). Thus, flag leaves seem to respond to the expected demand for amino acids before sink strength is established in grains and respectively, the transition phase starts two days earlier. Striking differences are visible between flag leaves and glumes during pre-anthesis and early grain development (−4 to 6 DAF). This strengthens the assumption that glumes function in a distinctive way compared to flag leaves, at least during early grain development.

Increasing N demand can generate long-distance signals within the plant [64]. Possibly certain N compounds or amino acids could be translocated through the phloem and its fluctuating levels might signal the nitrogen status of the plant. Especially glutamate has been suggested to function as an evolutionary conserved long-distance signal in plants as well as in animals [65]. Cytokinins can also be involved in signalling the N status of the plant [66,67]. The phloem might be important in delivering signals to distantly located plant organs. In this way, high grain demands for N might decrease assimilate levels in the phloem which could generate signals for remobilisation in the source.

We hypothesise that in such a way phases of grain developmen could be perceived in the ear-near tissues flag leaf and glumes. This would suggest development-specific signalling which mediates sink-source communication during grain development and which also might regulate *AAT* gene expression. Tissue-specific regulation of sink/source transition can also play a role as observed from fluctuating transcript abundances of *AAT* genes especially in flag leaves. Overall, such hypothetical relationship in sink-source communication has been derived from expression profiles of a collection of genes which transcripts are over-represented in a specific set of pyrosequences which demonstrates the power of this approach.

## Conclusions

Analysis of the overall dataset showed, that flag leaves and glumes obviously have different functions during early grain development when endosperm sink strength is low. Analogies in gene expression observed between glumes and the supplying maternal tissues indicate that glumes function as mediators between remobilising vegetative tissues and accumulating grains. Combination of already known and newly derived sequence information reduced redundancy, increased contig length and identified new members of cysteine peptidase and N transporter gene families. Participation of the respective gene products in either N remobilisation or accumulation can be expected. Amino acid permeases (AAPs), a sub-group of the AAT family of N transporters seem to be predominant in N retranslocation and grain filling. In phylogenetic trees, putative *HvAAP* genes which are highly expressed in remobilising tissues cluster together with functionally characterised Arabidopsis transporters responsible for long-distance transport of amino acids. In contrast, grain-specific AAPs are most similar to Arabidopsis transporters active in developing embryos. Based on expression profiling of *AAT* genes and subsequent statistical data analysis we hypothesise that high grain demands for N might decrease assimilate levels in the phloem which could generate signals for remobilisation in the source. Our future scientific work will be focussed on identification of metabolic/hormonal phloem components, which signal the grain N status to the plant.

Overall, cysteine peptidase and N transporter sequences as identified in this study might be of high interest for applied research because of their obvious role in N partitioning for grain filling. Up to now, information is based only on transcript data. For application of this knowledge in development of new breeding strategies, the specific role of individual candidates (for instance specific cysteine peptidases, LHT, AAP and ANT genes) in N remobilisation and accumulation has to be clarified.

## Methods

### Plant growth and RNA preparation

Barley (*Hordeum vulgare* L.) plants of cv. Barke were grown in pots with Substrat2 (Klasmann-Deilmann GmbH, Germany) and fertilized with 10 g Osmocote (Scotts Ind BV, Netherlands) and 0.2% solution of Hakaphos red (Compo GmbH & Co KG, Germany) at 3 leaf and at heading stage, respectively. The plants grew in the greenhouse at 18°C with 16 h of light. Developmental stages for barley grains were determined as described by Weschke et al. [63]. Flag leaves, glume fractions (including palea, lemma and awn) and grain tissues were collected based on grain developmental stages. For flag leaves and glumes, samples were collected in two day intervals starting from 4 days before anthesis until 24 days after flowering (DAF). Grains from 0, 4, 8, 10, 12, 14 DAF were manually dissected into maternal and filial parts and whole caryopses were sampled at 16, 20 and 24 DAF. Total RNA was isolated separately from each tissue at different stages using Purescript RNA isolation kit (Biozym, Hamburg, Germany). To prepare RNA samples for transcriptome sequencing, equal amounts of RNA from all stages were united for each tissue to achieve 27 μg of RNA for each organ. Pyrosequencing of the three libraries using the Roche/454 GS-FLX Titanium technology was done by GATC Biotech (Konstanz, Germany).

### Sequence analysis

Generated raw reads are accessible at EMBL/EBI, European Nucleotide Archive (ENA Project ERP001286, [68]). All reads were adaptor and quality trimmed using SeqClean [69]. Clustering and assembling was done separately for each library using the TGICL pipeline [70]. The pipeline uses megablast [71] for pre-clustering and CAP3 [72] for sequence assembly. The overlap settings for assembly were 95% identity and 35 bp overlap (all other parameters were set to default). The best BLASTn [73] hit of individual reads against all contigs was used to determine read numbers per contig. To cross-check these results, a second assembly has been generated using Newbler [74], (data not shown).

Comparisons of assembled sequences to public databases and each other were done by BLAST similarity searches [73] with different E-values. For stepwise blast, a perl script [75] using several BioPerl packages [76] was written. To determine Gene Ontology (GO) terms, sequences were analysed using Blast2GO [34,35].

To obtain *in silico* expression levels numbers of all single reads matching one contig in the BLASTn query (E-value >1E^-20^) and derived from the same tissue were summarized.

### Identification of N transporter sequences

BLASTn of all contigs from the tissue-specific assemblies was done against N transporter collections (AAT, OPT, NTR1/PTR) selected from H35 [30]. Setting blast E-value to <1E^-10^ we considered sequences beyond that cut-off as putative candidates. Candidates were assembled with H35 sequences using the standard algorithm of Lasergene 8 (DNAStar Inc, Madison, USA). Newly created contigs and remaining RNA-seq singletons were used for further analysis.

Confirmation of the amino acid sequences was done by BLASTp against Aramemnon [12] and comparison to rice homologs. Identities between 30-94% (AAT), 59-92% (OPT) and 51-94% (NRT1/PTR) were observed. To exclude contaminations, a BLASTn of these sequences was done against the available barley genomic sequence [41] and verified the barley specificity with identities between 97 and 100% on nucleotide level.

### Phylogenetic trees

Basic alignments for tree construction and corresponding sequence distance matrices were calculated using the ClustalW algorithm with Blosum protein weight matrix in Lasergene 8 (DNAStar Inc, Madison, USA) with HvPT1 [77] included as outgroup. For each dataset (AAT, OPT, and NRT1/PTR) mean pairwise distances between sequences were calculated and clustered with the neighbor-joining algorithm in PAUP* [78]. Bootstrap support values were calculated by 1000 bootstrap re-samples for each dataset. Phylogenetic trees were visualised with FigTree v1.3.1 [79]. The EMBL accessions of RNA-seq N transporter sequences and IPK previously unpublished sequences (HvAAP1, HvAAP2, HvPTR2, HvPTR3, HvPTR6) are summarised in Additional file 8: Table S3, the additionally used EST-sequences are available at H35 [30].

### Identification of cysteine peptidase sequences

Cysteine peptidases were identified by BLASTn searches (E-value <1E^-10^) against known cysteine peptidases sequences from H35 and by BLASTx (E-value <1E^-10^) searches against cysteine peptidases from *Arabidopsis thaliana* and *Oryza sativa*[80,81]. Candidates from the three tissue libraries were assembled with H35 sequences using the standard algorithm of Lasergene 8 (DNAStar Inc, Madison, USA). The created contigs and remaining RNA-seq singletons were used for further analysis. For annotation and classification of the cysteine peptidases the translated amino acid sequences were compared by BLASTp to those of known cysteine peptidases from barley and homologs from Arabidopsis and rice in the MEROPS database [37]. To exclude contaminations, sequences were blasted against the available barley genomic sequence [41] and were verified with identities between 93 and 100% at nucleotide level.

### qRT-PCR analysis

Plant material was collected in two day steps (flag leaf −4 – 24 DAF, glumes −4 – 24 DAF, filial grain tissue 4 – 24 DAF) and homogenised at −80°C. Total RNA was extracted using Spektrum Plant Total RNA Kit (Sigma Aldrich, Steinheim, Germany) and treated with RNase-free TURBO™ DNase (Ambion, Life Technologies, Darmstadt, Germany). cDNA was synthesized from 2 μg of total RNA with Superscript™ III (Invitrogen, Life Technologies, Darmstadt, Germany) using poly(dT) and random hexamer primers according to the manufacturer’s instructions. 1 μg diluted cDNA (1:32) was used for qRT-PCR with gene-specific primers (Additional file 9: Table S1). Real time PCR was performed using ABI Prism 7900HT Sequence Detection System and Power SybrGreen PCR Mastermix reagent; data was analysed with SDS 2.2.1 Software (all Applied Biosystems, Darmstadt, Germany). Determination of a suitable reference gene, test of PCR efficiencies and determination of CT values were done according to Radchuk et al. [82]. The CT values were determined for three biological replicates, with three technical replicates for each value, and normalized against *actin* expression (ΔCT). The arithmetic averages of the ΔCT values were calculated and 2^-ΔCT^ values were used for clustering and visualization of data with Multiple Experiment Viewer v4.7 [83].

### PCA and K-means clustering

The entire set of qRT-PCR data was subjected to principle component analysis (PCA) and analysed using the J-Express software 2011 [84]. Thereby, the first axis was placed in the direction of the largest variance component, the second orthogonal axis in direction of the second largest variance. As about 60% of the total variance is represented along the first axis, and more than 12% along the second, coordinates on these two axes which together represent nearly two third of the total variance are plotted in Figure 9. To center the given data set and to define number of clusters representative for each tissue K-means clustering [85] was performed using OriginPro 8.1 [86].

## Abbreviations

AAP: Amino acid permeases; AAT: Amino acid transporters; DAF: Days after flowering; ETC: Endosperm transfer cells; FL: Flag leaf; G: Grain; GL: Glumes; GS: Glutamine synthetase; H35: Assembly 35 of HarvEST:Barley v1.83 [30]; NP: Nucellar projection; NRT1/PTR: Nitrate/peptide transporters; OPT: Oligopeptide transporters; PCA: Principle component analysis; PS: Phytosiderophores.

## Competing interests

The authors declare that they have no competing interests.

## Authors’ contributions

SK: RNA preparation, analysis of qRT-PCR raw data, comparison between RNA-seq, H35 and Aramemnon data, alignment of N transporter sequences. JH, BS, TS, US: read quality trimming, sequence assembly and annotation, comparison to barley genome sequences. JH: alignment and annotation of cysteine peptidase sequences, clustering to the MEROPS clades. FRB: construction of phylogenetic trees. VR: RNA preparation. FA: qRT-PCR analysis. KK: critical revising of intellectual content. HW: substantial contribution to the concept, critical revising of intellectual content. WW: substantial contribution to the concept, interpretation of data. All authors read and approved the final manuscript.

## Supplementary Material

Additional file 1 **Figure S1.** Detailed phylogentic tree of plant AATs. Clustering of 63 Arabidopsis, 80 rice and 72 barley sequences (accounting for 59 unique sequences). To support the tree, barley full-length sequences from H35 and publications were included. Barley sequences are written in violet, functionally characterised transporters in dark green (see Additional file 2: Table S2). Click here for file

Additional file 2 **Table S2.** Functionally characterised N transporters in phylogentic trees. Click here for file

Additional file 3 **Figure S2.** Sequence distance matrices of AAT genes from Lasergene data. Only percent similarity is shown. Click here for file

Additional file 4 **Figure S3.** Detailed phylogentic tree of plant NRT1/PTRs. Clustering of 52 Arabidopsis, 81 rice,1 *Brassica napus*, 1 *Alnus glutinosa* and 52 barley sequences (accounting for 46 unique sequences). To support the tree, barley full-length sequences from H35 and publications were included. Barley sequences are written in violet, functionally characterised transporters in dark green (see Additional file 2: Table S2). Click here for file

Additional file 5 **Figure S4.** Sequence distance matrices of NRT1/PTR genes from Lasergene data. Only percent similarity is shown. Click here for file

Additional file 6 **Figure S6.** Sequence distance matrices of OPT genes from DNAStar data. Only percent similarity is shown. Click here for file

Additional file 7 **Figure S5.** Detailed phylogentic tree of plant OPTs. Clustering of 17 Arabidopsis, 26 rice and 24 barley sequences (accounting for 22 unique sequences). To support the tree, barley full-length sequences from H35 and publications were included. Barley sequences are written in violet, functionally characterised transporters in dark green (see Additional file 2: Table S2). Click here for file

Additional file 8 **Table S3.** EMBL accessions of N Transporters. Click here for file

Additional file 9 **Table S1.** Primers used in qRT-PCR. Click here for file
