# Supplementary material for: A putative role for amino acid permeases in sink-source communication of barley tissues uncovered by RNA-seq
Source: BMC Plant Biol. 2012 Aug 30;12:154. doi: 10.1186/1471-2229-12-154 (PMC3495740; doi:10.1186/1471-2229-12-154)
Supplement: Additional file 1 — Figure S1. Detailed phylogentic tree of plant AATs. Clustering of 63 Arabidopsis, 80 rice and 72 barley sequences (accounting for 59 unique sequences). To support the tree, barley full-length sequences from H35 and publications were included. Barley sequences are written in violet, functionally characterised transporters in dark green (see Additional file 2: Table S2). [file 1471-2229-12-154-S1.pdf]

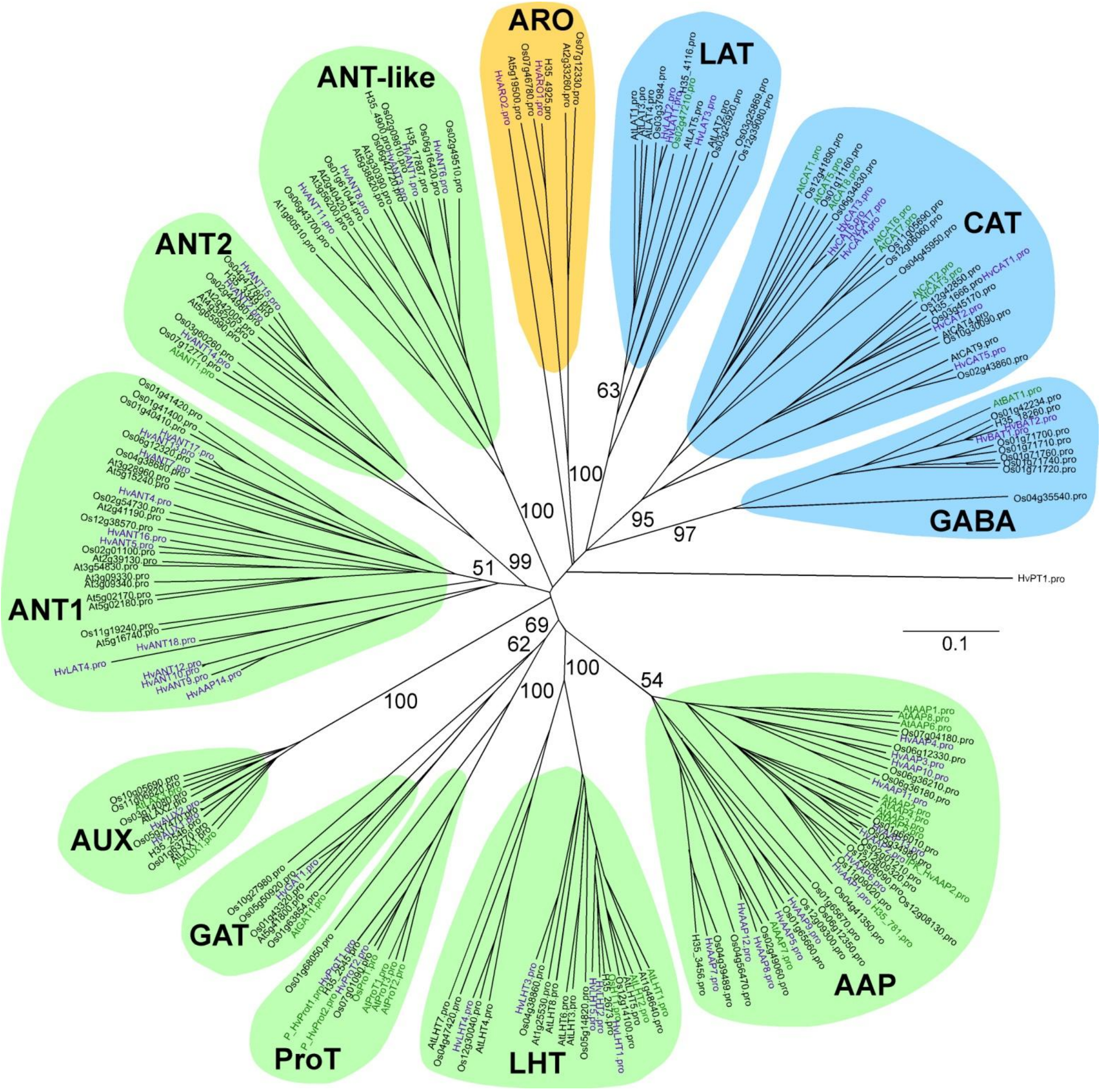

Additional Figure 1 Detailed phylogentic tree of plant AATs. Clustering of 63 Arabidopsis, 80 rice and 72 barley sequences (accounting for 59 unique sequences). To support the tree, barley full-length sequences from H35 and publications were included. Barley sequences are written in violet, functionally characterised transporters in dark green (see Additional Table 2).
