# Supplementary material for: A putative role for amino acid permeases in sink-source communication of barley tissues uncovered by RNA-seq
Source: BMC Plant Biol. 2012 Aug 30;12:154. doi: 10.1186/1471-2229-12-154 (PMC3495740; doi:10.1186/1471-2229-12-154)
Supplement: Additional file 2 — Table S2. Functionally characterised N transporters in phylogentic trees. [file 1471-2229-12-154-S2.pdf]

Additional Table 2 Functionally characterised N transporters in phylogentic trees

Substrate	Genefamily	Subfamily	Gene (Accession)	References
Amino Acids	ATF	AAP	AtAAP1 (At1g58360), AtAAP2 (At5g09220), AtAAP3 (At1g77380), AtAAP4 (At5g63850), AtAAP5 (At1g44100), AtAAP6 (At5g49630), AtAAP7 (At5g23810), AtAAP8 (At1g10010), HvAAP1(-), HvAAP2 (-),	**AtAAP1** Hsu et al., 1993, Frommer et al., 1993, Boorer et al., 1996, Chang et al., 1997, Lee et al., 2007; **AtAAP1-2** Kwart et al., 1993; **AtAAP1-6** Fischer et al., 2002; **AtAAP1-5** Fischer et al., 1995; **AtAAP1,5-8** Okumoto et al., 2002; **AtAAP2** Zhang et al., 2010; **AtAAP5** Boorer et al., 1997; HvAAP1,2 (unpublished)
		LHT	AtLHT1 (At5g40780), AtLHT2 (At1g24400), OsHT1 (Os08g03350)	**AtLHT1** Chen et al., 1997, Hirner et al., 2006, Svennerstam et al., 2007; **AtLHT2** Lee et al. 2004; **OsHT1** LIU et al., 2005
		ProT	AtProT1 (At2g39890), AtProT2 (At3g55740), AtProT3 (At2g36590), OsProT (Os03g44230), HvProT (BAB69951), HvProT2 (AB545851)	**AtProT1,2** Rentsch et al., 1996; **AtProT1-3** Grallath et al., 2005, Lehmann et al., 2011; **OsProT** Igarashi et al., 2000; HvProT Ueda et al., 2001; **HvProT2** Fujiwara et al., 2010
		GAT	AtGAT1 (At1g08230)	**AtGAT1** Meyer et al, 2006
		ANT	AtANT1 (At3g11900)	**AtANT1** Chen et al, 2001
		AUX	AtLAX1 (At5g01240), AtLAX3 (At1g77690)	**AtAUX1** Swarup et al., 2001 and 2004, Kleine-Vehn et al., 2006, Carrier et al., 2009, Blakeslee et al., 2007; **AtLAX3** Lee et al., 2011
	APC	CAT	AtCAT1 (At4g21120), AtCAT2 (At1g58030), AtCAT3 (At5g36940), AtCAT5 (At2g34960), AtCAT6 (At5g04770), AtCAT7 (At3g10600), AtCAT8 (At1g17120)	**AtCAT1** Frommer et al., 1995; **AtCAT1,2,3,5,6,8** Su et al., 2004; **AtCAT6** Hammes et al., 2006; **AtCAT8** Yang et al., 2010
		GABA	AtBAT1 (At2g01170)	**AtBAT1** Dundar et al., 2009
		LAT	OsPUT1 (Os02g47210)	**OsPUT1** Mulangi et al., 2011
Nitrate & Peptides	NRT1/PTR	I	AtNRT1.1 (At1g12110), BnNRT1.2 (Q43390), AgDCAT1 (Q7Y0R5), AtNRT1.2 (At1g69850), AtNRT1.4 (At2g26690), OsNRT1.3 (Os10g40600), OsSP1 (Os11g12740)	**AtNRT1.1** Huang et al., 1996, Lauter et al., 1996, Wang et al., 1998, 2009, Lejay et al., 1999, Liu et al., 1999, Guo et al., 2001, 2002, 2003, Orsel et al., 2002, Lejay et al., 2003, Loque et al., 2003, Okamoto et al., 2003, Munos et al., 2004, Krouk et al., 2006, Remans et al., 2006, Martin et al., 2008, Walch-Liu et al., 2008, Gojon et al., 2011, Castaings et al., 2011; **AtNRT1.2** Huang et al., 1999; **AtNRT1.4** Chiu et al., 2004; **BnNRT1.2** Zhou et al., 1998; **AgDCAT1** Jeong et al., 2004; **OsSP1** Li et al., 2009; **OsNRT1.3** Hu et al., 2006;
		II	AtNRT1.5 (At1g32450), AtNRT1.8 (At4g21680), AtPTR1 (At3g54140), AtPTR2 (At2g02043), AtPTR4 (At2g02020), AtPTR5 (At5g01180), AtPTR6 (At1g62200), OsPTR1 (Os07g01070), OsPTR4 (Os07g41250), OsPTR5 (Os04g50940), OsPTR6 (Os04g50950), OsPTR7 (Os01g04950), OsPTR8 (Os03g51050), HvPTR1 (BAJ95903)	**AtNRT1.5** Lin et al., 2008; **AtNRT1.8** Li et al., 2010; **AtPTR1** Dietrich et al., 2004; **AtPTR2** Song et al., 1997, Chiang et al., 2004; **AtPTR3** Karim et al., 2005, 2007; **AtPTR4,6** Weichert et al., 2011; **AtPTR1,5** Komarova et al., 2008, Hammes et al., 2010; **At1g68570** Sugiura et al., 2007; **OsPTR1-8** Ouyang et al. 2010; **HvPTR1** Waterworth et al., 2000, West et al., 1998;
		III	AtPTR3 (At5g46050), OsPTR3 (Os10g33210)	**AtPTR3** Karim et al., 2005, 2007; **OsPTR1-8** Ouyang et al. 2010;
		IV	AtNAXT1 (At3g45650), AtNRT1.6 (At1g27080), AtNRT1.7 (At1g69870), AtNRT1.9 (At1g18880), AtNRT1.11 (At1g52190), OsPTR2 (Os12g44100)	**AtNAXT1** Segonzac et al., 2007; **AtNRT1.6** Almagro et al., 2008; **AtNRT1.7** Fan et al., 2009; **AtNRT1.11** Nambara et al., 2009; **AtNRT1.9** Wang et al., 2011; **OsPTR1-8** Ouyang et al. 2010;
Oligopeptides	OPT		AtOPT1 (At5g55930), AtOPT3 (At4g16370), AtOPT4 (At5g64410), AtOPT5 (At4g26590), AtOPT6 (At4g27730), AtOPT7 (At4g10770), OsGT1 (Os06g03560)	**AtOPT1,4-7** Koh et al., 2002; **AtOPT3** Wintz et al., 2003; **AtOPT4** Osawa et al., 2006; **AtOPT6** Cagnac et al., 2004, Pike et al., 2009; **OsGT1** Zhang et al. 2004
Chelated Metals	YSL		AtYSL1 (At4g24120), AtYSL2 (At5g24380), AtYSL3 (At5g53550), OsYSL2 (Os02g43370), OsYSL6 (Os04g32050), OsYSL15 (Os02g43410), OsYSL18 (Os01g61390), HvYS1 (BAE71187.1), HvYS2 (BAL02122)	**AtYSL1** Le Jean et al., 2005; **AtYSL1,3** Waters et al., 2006, Chu et al., 2010; **AtYSL2** DiDonato et al., 2004, Schaaf et al., 2005; **OsYSL2** Koike et al., 2004, Ishimaru et al., 2010; **OsYSL6** Sasaki et al., 2010; **OsYSL15** Lee et al., 2009, Inoue et al. 2009; **OsYSL18** Aoyama et al., 2009; **HvYS1** Murata et al., 2006; **HvYS2** Araki et al., 2011

*Information regarding peptide transporters in NRT1/PTR and oligopeptide transporters in OPT families is given in green letters.
