# Supplementary material for: A putative role for amino acid permeases in sink-source communication of barley tissues uncovered by RNA-seq
Source: BMC Plant Biol. 2012 Aug 30;12:154. doi: 10.1186/1471-2229-12-154 (PMC3495740; doi:10.1186/1471-2229-12-154)
Supplement: Additional file 3 — Figure S2. Sequence distance matrices of AAT genes from Lasergene data. Only percent similarity is shown. [file 1471-2229-12-154-S3.pdf]

Amino acid transporters

		ATF group								ARO	APC group		
		AAP	ANT1	ANT2	ANT3	LHT	ProT	GAT	AUX		LAT	BAT	CAT
ATF group	AAP	54,9	17,3	16,9	14,0	25,5	21,6	24,0	16,0	8,3	9,2	8,8	9,0
	ANT1	***	41,4	24,3	17,7	18,8	16,5	17,8	15,8	8,4	9,8	8,4	9,8
	ANT2		***	52,5	18,2	17,1	17,0	18,0	15,5	8,6	9,3	7,9	10,1
	ANT3			***	54,2	14,7	15,2	14,5	12,0	8,1	9,1	7,7	8,9
	LHT				***	53,4	21,6	25,1	16,3	8,4	9,2	7,3	9,9
	ProT					***	68,5	27,6	13,7	8,2	10,2	8,2	9,4
	GAT						***	51,3	19,7	7,7	9,1	8,4	10,2
	AUX							***	78,0	7,5	8,0	8,3	9,1
ARO									***	37,4	8,5	8,2	8,8
APC group	LAT									***	48,8	12,9	13,9
	BAT										***	67,5	12,2
	CAT											***	39,0

Additional Figure 2 Sequence distance matrices of *AAT* genes from Lasergene data. Only percent similarity is shown.
