# Supplementary material for: A putative role for amino acid permeases in sink-source communication of barley tissues uncovered by RNA-seq
Source: BMC Plant Biol. 2012 Aug 30;12:154. doi: 10.1186/1471-2229-12-154 (PMC3495740; doi:10.1186/1471-2229-12-154)
Supplement: Additional file 5 — Figure S4. Sequence distance matrices of NRT1/PTR genes from Lasergene data. Only percent similarity is shown. [file 1471-2229-12-154-S5.pdf]

NRT1/PTR transporters

		subgroup according to Tsay et al			
		**I**	**II**	**III**	**IV**
**subgroup according to Tsay et al**	**I**	39,1	33,5	32,1	30,3
	**II**	***	44,6	34,6	32,0
	**III**		***	42,3	30,1
	**IV**			***	37,9

Additional Figure 4 Sequence distance matrices of *NRT1/PTR* genes from Lasergene data. Only percent similarity is shown.
