# Supplementary material for: A putative role for amino acid permeases in sink-source communication of barley tissues uncovered by RNA-seq
Source: BMC Plant Biol. 2012 Aug 30;12:154. doi: 10.1186/1471-2229-12-154 (PMC3495740; doi:10.1186/1471-2229-12-154)
Supplement: Additional file 6 — Figure S6. Sequence distance matrices of OPT genes from DNAStar data. Only percent similarity is shown. [file 1471-2229-12-154-S6.pdf]

**OPT transporters**

| | OPT | YSL total | YSL1 | YSL2 | YSL3 | YSL4 |
|---|---|---|---|---|---|---|
| OPT | 56,8 | 12,5 | 13,0 | 11,4 | 12,6 | 12,2 |
| YSL total | | 52,8 | 48,7 | 50,0 | 51,2 | 41,2 |
| YSL1 | | | 69,4 | 51,4 | 53,3 | 39,7 |
| YSL2 | | | | 73,0 | 55,8 | 39,0 |
| YSL3 | | | | | 70,9 | 44,1 |
| YSL4 | | | | | | 45,6 |

**Additional Figure 6 Sequence distance matrices of *OPT* genes from DNAStar data.** Only percent similarity is shown.
