# Supplementary material for: A putative role for amino acid permeases in sink-source communication of barley tissues uncovered by RNA-seq
Source: BMC Plant Biol. 2012 Aug 30;12:154. doi: 10.1186/1471-2229-12-154 (PMC3495740; doi:10.1186/1471-2229-12-154)
Supplement: Additional file 8 — Table S3. EMBL accessions of N Transporters. [file 1471-2229-12-154-S8.pdf]

Additional Table 3 EMBL accessions of N Transporters

EMBL accessions	Transporter name	Data source
HE820742	HvAAP1	IPK EST collection
HE820743	HvAAP2	IPK EST collection
HE858194	HvPTR2	IPK EST collection
HE858195	HvPTR3	IPK EST collection
HE858196	HvPTR6	IPK EST collection
HE965462	HvAAP1-5	RNA-seq
HE965463	HvAAP1-3	RNA-seq
HE965464	HvAAP2-3	RNA-seq
HE965465	HvAAP3	RNA-seq
HE965466	HvAAP4	RNA-seq
HE965467	HvAAP5	RNA-seq
HE965468	HvAAP6	RNA-seq
HE965469	HvAAP7	RNA-seq
HE965470	HvAAP8	RNA-seq
HE965471	HvAAP9	RNA-seq
HE965472	HvAAP10	RNA-seq
HE965473	HvAAP11	RNA-seq
HE965474	HvAAP12	RNA-seq
HE965475	HvAAP13	RNA-seq
HE965476	HvAAP14	RNA-seq
HE965477	HvANT1	RNA-seq
HE965478	HvANT2	RNA-seq
HE965479	HvANT3	RNA-seq
HE965480	HvANT4	RNA-seq
HE965481	HvANT5	RNA-seq
HE965482	HvANT6	RNA-seq
HE965483	HvANT7	RNA-seq
HE965484	HvANT8	RNA-seq
HE965485	HvANT9	RNA-seq
HE965486	HvANT10	RNA-seq
HE965487	HvANT11	RNA-seq
HE965488	HvANT12	RNA-seq
HE965489	HvANT13	RNA-seq
HE965490	HvANT14	RNA-seq
HE965491	HvANT15	RNA-seq
HE965492	HvANT16	RNA-seq
HE965493	HvANT17	RNA-seq

EMBL accessions	Transporter name	Data source
HE965494	HvANT18	RNA-seq
HE965495	HvAUX1-5	RNA-seq
HE965496	HvAUX2	RNA-seq
HE965497	HvARO1	RNA-seq
HE965498	HvARO2	RNA-seq
HE965499	HvCAT1	RNA-seq
HE965500	HvCAT2	RNA-seq
HE965501	HvCAT3	RNA-seq
HE965502	HvCAT4	RNA-seq
HE965503	HvCAT5	RNA-seq
HE965504	HvCAT6	RNA-seq
HE965505	HvCAT7	RNA-seq
HE965506	HvBAT2	RNA-seq
HE965507	HvBAT3	RNA-seq
HE965508	HvGAT1	RNA-seq
HE965509	HvLAT1	RNA-seq
HE965510	HvLAT2	RNA-seq
HE965511	HvLAT3	RNA-seq
HE965512	HvLAT4	RNA-seq
HE965513	HvLHT1	RNA-seq
HE965514	HvLHT2	RNA-seq
HE965515	HvLHT3	RNA-seq
HE965516	HvLHT4	RNA-seq
HE965517	HvLHT5	RNA-seq
HE965518	HvProT1	RNA-seq
HE965519	HvProT2	RNA-seq
HE965520	HvOPT1	RNA-seq
HE965521	HvOPT2	RNA-seq
HE965522	HvOPT3	RNA-seq
HE965523	HvOPT4	RNA-seq
HE965524	HvOPT5	RNA-seq
HE965525	HvOPT6	RNA-seq
HE965526	HvOPT7	RNA-seq
HE965527	HvOPT8	RNA-seq
HE965528	HvOPT9	RNA-seq
HE965529	HvOPT10	RNA-seq
HE965530	HvOPT11	RNA-seq
HE965531	HvYSL1	RNA-seq
HE965532	HvYSL2-5	RNA-seq

EMBL accessions	Transporter name	Data source
HE965533	HvYSL2-3	RNA-seq
HE965534	HvYSL3	RNA-seq
HE965535	HvYSL4	RNA-seq
HE965536	HvYSL6	RNA-seq
HE965537	HvYSL7-5	RNA-seq
HE965538	HvYSL7-3	RNA-seq
HE965539	HvYSL8-5	RNA-seq
HE965540	HvYSL8-3	RNA-seq
HE965541	HvYSL9	RNA-seq
HE965542	HvYSL10	RNA-seq
HE965543	HvYSL11	RNA-seq
HE965544	HvYSL12	RNA-seq
HE965545	HvNRT1(PTR)I-1	RNA-seq
HE965546	HvNRT1(PTR)I-2	RNA-seq
HE965547	HvNRT1(PTR)I-3-5	RNA-seq
HE965548	HvNRT1(PTR)I-3-3	RNA-seq
HE965549	HvNRT1(PTR)I-4-5	RNA-seq
HE965550	HvNRT1(PTR)I-4-3	RNA-seq
HE965551	HvNRT1(PTR)I-5	RNA-seq
HE965552	HvNRT1(PTR)I-6	RNA-seq
HE965553	HvNRT1(PTR)I-7	RNA-seq
HE965554	HvNRT1(PTR)I-8	RNA-seq
HE965555	HvNRT1(PTR)II-1-5	RNA-seq
HE965556	HvNRT1(PTR)II-1-3	RNA-seq
HE965557	HvNRT1(PTR)II-2	RNA-seq
HE965558	HvNRT1(PTR)II-3-5	RNA-seq
HE965559	HvNRT1(PTR)II-3-3	RNA-seq
HE965560	HvNRT1(PTR)II-4	RNA-seq
HE965561	HvNRT1(PTR)II-5	RNA-seq
HE965562	HvNRT1(PTR)II-6	RNA-seq
HE965563	HvNRT1(PTR)II-7	RNA-seq
HE965564	HvNRT1(PTR)II-8	RNA-seq
HE965565	HvNRT1(PTR)II-9	RNA-seq
HE965566	HvNRT1(PTR)II-10	RNA-seq
HE965567	HvNRT1(PTR)II-11	RNA-seq
HE965568	HvNRT1(PTR)II-12	RNA-seq
HE965569	HvNRT1(PTR)II-13	RNA-seq
HE965570	HvNRT1(PTR)II-14-5	RNA-seq
HE965571	HvNRT1(PTR)II-14-3	RNA-seq

EMBL accessions	Transporter name	Data source
HE965572	HvNRT1(PTR)II-15	RNA-seq
HE965573	HvNRT1(PTR)II-16	RNA-seq
HE965574	HvNRT1(PTR)II-17	RNA-seq
HE965575	HvNRT1(PTR)III-1-5	RNA-seq
HE965576	HvNRT1(PTR)III-1-3	RNA-seq
HE965577	HvNRT1(PTR)III-2	RNA-seq
HE965578	HvNRT1(PTR)III-3	RNA-seq
HE965579	HvNRT1(PTR)III-4-5	RNA-seq
HE965580	HvNRT1(PTR)III-4-3	RNA-seq
HE965581	HvNRT1(PTR)III-5-5	RNA-seq
HE965582	HvNRT1(PTR)III-5-3	RNA-seq
HE965583	HvNRT1(PTR)III-6-5	RNA-seq
HE965584	HvNRT1(PTR)III-6-3	RNA-seq
HE965585	HvNRT1(PTR)III-7	RNA-seq
HE965586	HvNRT1(PTR)III-8	RNA-seq
HE965587	HvNRT1(PTR)III-9	RNA-seq
HE965588	HvNRT1(PTR)III-10	RNA-seq
HE965589	HvNRT1(PTR)III-11	RNA-seq
HE965590	HvNRT1(PTR)III-12	RNA-seq
HE965591	HvNRT1(PTR)III-13	RNA-seq
HE965592	HvNRT1(PTR)III-14	RNA-seq
HE965593	HvNRT1(PTR)IV-1-5	RNA-seq
HE965594	HvNRT1(PTR)IV-1-3	RNA-seq
HE965595	HvNRT1(PTR)IV-2	RNA-seq
HE965596	HvNRT1(PTR)IV-3	RNA-seq
HE965597	HvNRT1(PTR)IV-4	RNA-seq
HE965598	HvNRT1(PTR)IV-5	RNA-seq
HE965599	HvPTR1-5	RNA-seq
HE965600	HvPTR1-3	RNA-seq
HE965601	HvPTR1-like_1	RNA-seq
HE965602	HvPTR1-like_2	RNA-seq
HE965603	HvPTR1-like_3	RNA-seq
HE965604	HvPTR1-like_4	RNA-seq
HE965605	HvPTR1-like_5	RNA-seq
HE965606	HvPTR1-like_6	RNA-seq
HE965607	HvPTR1-like_7	RNA-seq
HE965608	HvPTR1-like_8	RNA-seq
HE965609	HvPTR1-like_9	RNA-seq
HE965610	HvPTR1-like_10	RNA-seq

EMBL accessions	Transporter name	Data source
HE965611	HvPTR1-like_11	RNA-seq
HE965612	HvPTR1-like_12	RNA-seq
HE965613	HvPTR1-like_13	RNA-seq
HE965614	HvPTR1-like_14	RNA-seq
HE965615	HvPTR1-like_15	RNA-seq
HE965616	HvPTR1-like_16	RNA-seq
HE965617	HvPTR1-like_17	RNA-seq
HE965618	HvPTR1-like_18	RNA-seq
HE965619	HvPTR1-like_19	RNA-seq
HE965620	HvPTR1-like_20	RNA-seq
HE965621	HvPTR1-like_21	RNA-seq
HE965622	HvPTR1-like_22	RNA-seq
HE965623	HvPTR1-like_23	RNA-seq
HE965624	HvPTR1-like_24	RNA-seq
HE965625	HvPTR1-like_25	RNA-seq
HE965626	HvPTR1-like_26	RNA-seq
