# Supplementary material for: A putative role for amino acid permeases in sink-source communication of barley tissues uncovered by RNA-seq
Source: BMC Plant Biol. 2012 Aug 30;12:154. doi: 10.1186/1471-2229-12-154 (PMC3495740; doi:10.1186/1471-2229-12-154)
Supplement: Additional file 9 — Table S1. Primers used in qRT-PCR. [file 1471-2229-12-154-S9.pdf]

Additional Table 1 Primers used in qRT-PCR

Gene	forward (5´-3´)	reverse (5´-3´)
Actin	GTGGATCTCGAAGGGTGAGT	AGCACTTCCGGTGGACAAT
HvAAP2	TCGGCGCCATCGGGTTCT	CGGCGGCAGCAATGGTGA
HvCAT1	TTAAAGCGGCGCAAAACAGC	AAGCCGAAGCCGTCAGGATTAG
HvANT1	TGCCGTGGTTTTTGTTGTCATC	TCCGCGGTTCCTTCTATCAGTC
HvAro2	GTGGGGATGGGGGTGTTCTCTG	TGCAGGGGGTCTCCGTTTACAA
HvCAT5	ACCCGCCTCGTTTTTCTTGTCC	ACCCCCACAGCGATGAGACTAA
HvProT1	GGCGCTCAGCACATTCCCTCTC	GCGGCGATAGCAGCGGTGAT
HvAUX1	CGCCGCTCTACTTCGTGTGG	GAGGGCGCGGAGGCAGAC
HvLHT1	GCCTCCGCGCTCTGCTACTC	CGGCCGGCCTTGTCTTGA
HvANT4	TAATGCCCGCCTTGTGCTTCTT	CTGCGTGCGTGTGGCTTTATTC
HvANT2	GGCCGCGGACAAGAAGAAGT	GTACCCCATGACACCGAACAGC
HvAAP3	TCGCCGCAGTCATGTCCTTCTC	TCAGTGCCGCCCATTGTTGTCT
HvANT5	CCGCCGCTGTGATGGGATAC	TGACTTCGCGGCAGCAACTCTT
HvLAT1	TCATTGCCGCGGAGAACTACCT	AAGCCAGCACCATCACCACGAC
HvANT3	CGCGCAATGAAATACGAGATGA	ACTGCCCCGGAGAATGAAGC
HvAro1	AGCGCCTCTTCTCCAATGTCAA	ATACTCCCTGGCTCGTGCTTCA
HvProT2	GGATACCGCGCACCAGATTAGC	GGAGGCGACCAAGCAGAGCA
HvAAP4	TTTGGCCGCTCACCGTCTACTT	GAGCCCCTGCACCGAACCA
HvAAP5	GATACGCGGCATTTGGTTCAGA	GGGGGCCGTCAGTATGTTGC
HvAAP6	TCGGCAACGTGGTGGGACTT	GCCGCGCTGCCTGATGTA
HvAAP1	GCAACATCGCCTTCGCCTACT	TTATCCCCGTCGCCTTCTTCAT
HvLHT2	ATCATCGCCGCCTGCTACTTCC	TCAAACACCGGCATCGCATAAA
HvANT6	GCCGCTGCTCCCGACGAA	GCCGACGATGGTGGTGGACAG
HvLHT4	AACGGCCGGCAGCAATCTCTTA	GGCCCCGCGCCACATAGG
HvANT17	AGAGCTGCTGCCTCCGAATAGA	CGCCACAAACATGGTGAGAAGA
HvAAP7	CAGGGGGTCATCAGCCAGAAGC	ATGCTCCCCGATCGCTCTAACC
